# On Hallucinations in Their Relation to Medical Jurisprudence

**Published:** 1861-10

**Authors:** A. Brierre de Boismont


### ART. XI. — ON HALLUCINATIONS IN THEIR RELATION TO MEDICAL JURISPRUDENCE.

By A. BRIERRE DE BOISMONT.

[THE following article is a translation of the terminal chapter in the third edition of M. Brierre de Boismont's work on Hallucinations,* just published. On the merits of this classical and widely-known work it would be idle for us to dilate. In the present edition it has been entirely re-written and re-cast, and its value proportionately increased. We have thought that we should most gratify our readers, as well as best indicate the nature of the modifications which the work has undergone, and its augmented excellence, by translating certain portions. We are induced to select, first, the chapter on the medico-legal relations of hallucinations, because the author has himself published it in a separate form, with certain additions (chiefly consisting in the illustrative cases being detailed at greater length), in the last number of the *Annales d'Hygiène Publique*.—ED.]

A PHENOMENON demonstrated in about three-fourths of our observations—since we have noted it 725 times in 1146 cases—the distinctive character of which is to convince of its reality those who experience it, and compel them to accord more confidence to the creations of the imagination than to the impressions furnished by the senses, necessarily presents some interesting subjects of medico-legal study. It is easy to conceive that the conscience and free-will have no longer then, as in the conception of delirium, the same chance of successfully struggling against error. The senses, subjugated by false impressions, which are com-

---

* *Des Hallucinations, sur Histoire Raisonnée des Apparitions, des Visions, de l'Extase, des Rêves, du Magnetisme, et du Sonnambulisme.* Par A. Brierre de Boismont. Troisième Edition, entirèment refondue. Paris: 1862.

pletely identical with them, have no power over the insane, who almost invariably replies to objections by these words of Bayle's patient:—"How are we to judge things? By the impression they produced. Well, I believe in the existence of demons, because I have seen, heard, touched, and felt them."

The surest means of studying hallucinations for legal purposes is to pass in review the symptoms of the principal forms of insanity, because they show us the direction of ideas and the line of conduct into which the insane are carried away.

The importance of this study has already been indicated in the chapter occupied with the description of hallucinations associated with the principal types of insanity, and in the different cases contained in our work. People begin to understand now that many of those extraordinary actions that were unhesitatingly set down to the account of crime ought to have been attributed to madness, and especially to hallucination. This opinion will become a truth when the conviction is established that independently of fantastic images, forms of persons and objects undergo the most astonishing metamorphoses, that voices exercise a despotic power, and irresistibly compel the will to obey their orders, &c.

Such a subject is of too much interest not to have a special chapter to itself. We will examine, 1st, the influence of hallucinations upon the conduct, sleeping and waking. 2nd. We will afterwards discuss to what extent hallucinations necessitate sequestration, suspension of civil rights, and whether this disposition of mind always nullifies civil acts.

We have already said that the starting-point of this subject ought to be an examination of the symptoms which have a close connexion with legal questions, and we shall confine our investigations within that circle.

In acute delirium, the violence of the symptoms, the continuousness of the intellectual disorder, the incoherence, and the confusion, render it difficult to separate hallucinations from illusions. Abandoned without control to external impressions and internal sensations, the brain is forced to submit to them and be mastered by them, the judgment being wholly wanting. Out of 32 cases of acute delirium 25 were complicated by hallucinations and illusions: the others were so disordered that it was impossible to inquire into them.

This kind of febrile maniacal madness by the intensity of the general agitation confuses all the brain; ideas take bodily shape, objects undergo a metamorphosis, and the mind lives in the midst of a continual phantasmagoria.

The hallucinations and illusions in this disease are the accessories of a delirium nearly always general, in the midst of which it is by no means rare to see a predominating delirious conception; but the mobility, confusion, and clashing of ideas often render difficult the observation of these sensorial perceptions. It is undoubtedly one of the forms of insanity in which they are most fugitive. The impressions they produce upon the mind are often painful; every form seems that of an enemy or of a demon, voices utter threatening words; beverages have a filthy flavour, or are poisoned. Five of these lunatics, under the influence of such sad impressions, attempted suicide, and two rushed upon persons present to do them an injury.

It is to this sad condition of the mind that must be attributed the suicides so frequently resulting from what are called cerebral or ardent fevers, and which are generally only cases of acute delirium.

Persons of this class ought to be the object of constant surveillance, owing to the variety, the mobility, and the suddenness of their illusions, which are more common than hallucinations.

The agitation of the *maniac*, his want of attention, the mobility of his ideas, are so many circumstances which interfere with the observation of sensorial impressions in this form of madness. Nevertheless, the longer interval between the intermittences, the species of connexion faintly visible between the incoherence of the conversation and the extravagance of the actions, the frequent presence of the patient, allow the disorders of the senses to be studied more attentively than in acute delirium.

The observations we have made upon mania amount to 229, and those complicated with hallucinations and illusions number 178 cases; there remain 51, which did not exhibit false sensorial perceptions.

The 178 facts are thus classified:—

| | |
|---|---|
| Hallucinations . . . . . . . . . . . . . . | 54 |
| Hallucinations and illusions . . . . . . . . . | 64 |
| Illusions . . . . . . . . . . . . . . . . | 60 |
| | 178 |

Hallucinations and illusions of mania give occasion to important observations. Marc, in his *Traité de la Folie Judiciaire,* strongly directed attention to this subject. It may be said, he affirms, that the majority of the extravagant, singular, reprehensible, dangerous, and criminal actions of the insane, arise, in the greater number of cases where they appear inexplicable, from hallucinations and hidden illusions.*

Out of 178 persons who presented this complication, 30—under the influence of false sensations—threatened death, struck,

* Caspar, *Traité Pratique de Médecine Légale.* 1861. 1° vol.]

overturned, wounded their pretended enemies, attempted to kill themselves, and if deplorable accidents did not take place, it was simply because they were promptly placed under restraint.

Hallucinatory perceptions and illusions of hearing lead to quarrels, to extreme anger and fury, and to violence, in considerable proportions. One of our patients, to whom insulting words were addressed, flew each time into a violent rage; he exclaimed that there had been enough of it, and that he must kill somebody. This patient is all the more dangerous because his attacks are instantaneous. Were he not constantly accompanied by his servant, some accident would have occurred; yet, despite his excitement, he knows what he is doing. However strict may be the surveillance, these auditory illusions constantly occasion struggles between the insane, and more or less serious injuries. A merchant used to hear two voices; one polite, the other insulting. With the former he was amiable, cheerful, ready to oblige; but when it was the turn of the latter, he became formidable—his strength, already great, was doubled. During one of his crises he seized, in an instant, a stake, and he had to be surrounded before it could be taken from him. Life is often endangered by this kind of illusion. Two ladies unexpectedly flew at a female employed on the establishment, and attempted to murder her; a vigorous struggle became necessary. A patient, insulted by these voices, threw himself out of the window. We attended once a merchant, in whose ears the word *bankruptcy* continually resounded. He energetically protested against this insult, and would have committed suicide had not precautions been taken.

We are certain that this kind of illusion, like that of the hearing, might have been the cause of quarrels, of violence, and of duels.

It has been seen in the chapter upon the hallucinations of mania, with what frequency persons are metamorphosed in the eyes of the insane; for, out of 124 cases, we have witnessed this change 62 times. This false sensation leads to deplorable consequences. A person, a prey to this delusion, flew at a friend, whom he took for a thief, knocked him down, thrashed him soundly, and called him a scoundrel. In our establishments, patients are often seen who try to beat other inmates whom they consider enemies. We attended a maniac who, believing himself surrounded by malignant beings, continually wished to rip up his companions. Many of those confided to us had struck policemen and others, because they had assumed the form of enemies: for the same reason some lunatics beat their keepers and severely wound them. One had his face mutilated by a decanter; when assistance came he was blinded by blood, and could not defend himself. Another keeper received upon the head a blow from an iron bar that a maniac had torn from the window of his cell; for some moments his life was despaired of.

The number of accidents is diminished by the fact that the forms seen are those of imaginary beings, who, having no living basis, excite the rage of the insane, and lead them into fantastic struggles, but occasion no disaster. A lunatic believed himself assailed by little demons brought in the air; they tortured him, poisoned his food, disseminated infectious smells, injured his eyes, shouted in his ears, and pinched his skin. At first he tried to exorcise them; but as his prayers were useless, he became furious, slashed at them with everything that came to his hand, and did not cease until exhausted by his superfluous efforts. If imagination had substituted human forms for these demons, murder might have occurred, as in many other cases.

The hallucinations and illusions of touch are also the starting-point of complaints, of recriminations, of acts of violence. The lunatic who imagines he has been struck is only too ready, if irritable and excitable, to give back, at some slight inconvenience to himself, the blows he thinks he has received. With women, sexual illusions demand the utmost attention. One of our patients declared she had been violated by the physician of the establishment, a political refugee—a man of the most honourable character, and who had an invincible repugnance to the insane. This patient made several complaints to me on the subject. The idea had seized upon her mind; she believed in it firmly. A year after quitting the establishment she came to me to repeat her denunciation; she was with a person who appeared to have taken the matter seriously. When they had related the facts to me, and I understood the object of their visit, I declared to them that I had no explanation to give, and referred them to the Procureur-Impérial, insisting upon this point: that an affair of such a nature did not allow of compromise, and that justice alone could decide it. The matter went no further. It is necessary to be on one's guard against these accusations, for though imaginary, they are not the less disagreeable for all that. The best plan is never to see young women, except in the presence of a servant; and when they have erotic instincts, they ought not to be visited or spoken with except in the presence of other patients.

The illusions of smell and taste often make maniacs suppose that their food and drink are poisoned, that they contain arsenic. Food and drink are therefore refused. Several of our patients, under the influence of this idea, have attempted suicide.

The general character of sadness, terror, despair, &c., found in the hallucinations and illusions of *melancholy monomania*, only too often foreshadow deplorable consequences. Out of 303 observations, which form our total returns of this kind of madness, we have noted 248 cases of hallucinations and illusions, of which 212 presented the most morbid indications of sorrow: suicide, it will thus be seen, is the accident most to be dreaded.

The actions resulting from these painful sensations may be divided into two classes: 1st, attempts at suicide; 2nd, attempts against others. Authors who have affirmed that suicide was always an act of madness take their arguments from lypemania (melancholic monomania). This form of delirium, which constitutes one of the most determined types of mental derangement, presents out of its 303 cases 170 instances of ideas of or attempts at suicide, and 118 refusals of food. We take into account here only 248 cases of melancholy madness with hallucinations and illusions. The two classes indicated are thus subdivided:—

| | |
|---|---|
| 1st. Attempts at suicide . . . . . . . . | 144 |
| 2nd. Attempts against others. . . . . . | 52 |

This proportion is considerable, since for the suicides it exceeds half of the total (1·72), and for the attempts against others it reaches nearly the fifth (4·76).

The motives given by the hallucinated for attempting suicide spring mostly from the painful sensations which oppress them. They unceasingly hear the voices of their enemies, of malignant persons who overwhelm them with reproaches, insults, threats, accuse them of having committed crimes, frightful sins, of being dishonoured, ruined, lost. One whom these reproaches drove to despair asked us to let his servant be always with him, for fear he should do himself an injury. One day, when the servant's back was turned, the patient dashed himself head foremost against the looking-glass that he smashed to atoms, falling senseless upon the floor, his skin torn in several places, and a small artery opened. Attended to and consciousness restored, he told us he had seen in the glass two dogs ready to devour him, and to escape this torment he had determined to finish with it at once. This spontaneity of determination is often so rapid that it can be neither foreseen nor prevented.

A young lady, to whose case we have already referred in the symptomatic exposition of hallucinations and illusions of acute delirium, was admitted into my establishment for acute delirium, with repeated attempts at suicide. Two keepers were placed by her side, and never quitted her. She had been to bed about two hours, and was very quietly conversing with the female inspector, when, suddenly seizing the cord of the curtains, she tied it so tightly round her throat, burying her head at the same time under the pillows, that for an instant the worst was feared, and for several days she bore the marks of the cord.

While rendering justice to the *non-restraint* system of the eminent Dr. Conolly, we have found it impossible of application in cases of this kind; and we would cite the case of a young person who, treated according to his principles, found means to obtain possession of a piece of glass, made a jagged wound in her arm near the vessels, and was sent away from the establishment that its method might not be deviated from, and because it was feared that some unfortunate occurrence might happen. If the strait-waistcoat had been employed—despite the supplications of relatives—in an establishment under my inspection, upon the entrance there of a female patient who had permanent suicidal mania, and to whom I had allotted a special attendant, I should not have been cited before a tribunal for a lunatic suicide, a circumstance without precedent until then, and I should have avoided a demand for damages that I might have set aside, but which I preferred to satisfy, in accordance with the fashion of the day. Nevertheless, it may fairly be stated that the sword of Damocles is suspended above the heads of directors of lunatic asylums.

A vision may at any moment provoke this thought. We have related one example; here is a second. Talking with a lady suffering from hallucinations, she told us she had just seen her funeral pass, and added, "But for the fear of scandal, I should have made the illusion a reality." The suicide in this case could not have been foreseen, and the determining cause would have remained unknown.

Dr. Baumes, medical director of the asylum of Quimper, states in one of his reports that a man was brought into his establishment in consequence of a sudden hallucination, which had had a deplorable result. All on a sudden a voice cried out to him, "Kill your wife," and he instantly shot her dead with a pistol. Scarcely was the act committed than the hallucination disappeared, and he no longer had delirium. An investigation took place, and the feeling was strongly against the murderer. Doubts existed, however, and a medical examination was ordered. The jury decided, despite the Procureur-Impérial, that the accused was not in a responsible state. He was sent to the Quimper Asylum. For a year the most minute examination discovered neither hallucination nor delirium; when, at a moment in which it was least expected, he jumped from a second storey and dislocated his left shoulder. Just before a voice had been saying to him, "Throw yourself out." When we saw this patient, in passing through Quimper, he was once more calm.* It is evi-

* A. Brierre de Boismont, "Une Visite en Bretagne à l'asile Saint-Athanase; quelques mots sur la Vie à l'air libre."—*Union Médicale*, note 403. 1857.

dent that, in this case, the character of duration that Dr. Brossius considers fundamental was entirely wanting in the hallucination: we think with M. Renaudin that this phenomenon in analogous circumstances is the product of various modifications of the somatic state, and in particular of the general susceptibility which constitutes its gravity and irresistibility.*

Every one must have remarked that painful and melancholy ideas sometimes suddenly arise in the mind, and remain there with strong persistence. You drive them out; they return. We knew a man who fancied that his house was going to catch fire; he was astonished at this idea, and could not divine its origin; it ceased, reappeared, tormented him for several days, especially at night, compelling him then to rise, disappeared, and showed itself afterwards at long intervals. It has existed for some years: he takes it for what it is, no longer troubles himself about it; but he has observed that it grows more intense when he is put out, and when he is not very well. This is not an isolated fact, and we do not hesitate to say that it prevails among many people. This singular state is especially common to nervous people. If by any course the idea is not repelled, it chooses its abiding place, and may perhaps subject the organization to its despotic power. The irresistibility of certain ideas is proved by a thousand examples. Every medical man has heard the hallucinated declare, "I am compelled to do that; a voice commands me to strike." It is unquestionable that these false impressions form a marked proportion in the statistics of suicide collected every year by the administration of justice.

The suddenness of the hallucinations is sufficiently established by the examples we have just cited. The annals of science contain many others. When they do not occasion extravagant acts, they fail to fix the attention so much; they even pass unperceived, or are observed only in special establishments. But when they are the cause of a crime, judgment should not be hasty; all the particulars which may throw a light on the circumstance ought, on the contrary, to be examined. There are some hallucinations, like homicidal impulses, which exhibit themselves unexpectedly, without having been announced by any derangement of the mind. Knowledge of antecedents may often point to madness; singularities and eccentricities augur badly for the integrity of the reason; assumption becomes stronger if some strange act at a previous period can be ascertained. Changes of disposition and of demeanour are of extreme value; investigations into hereditary character in such circumstances are also very useful; the motives of action, the replies of the person inculpated, should be

---

* *Annales Medico-Psychologiques*, Janvier, 1854, 3e series, t. ii. p. 109.

the object of serious examination. When the person questioned replies that he obeyed a voice; that he was irritated by insults unceasingly bestowed upon him; that he wished to be revenged upon his persecutors—then, if the victim was unknown to him, or if he had been on good terms with that victim, and if it is found that no connexion of any kind exists between them, assumption is of still greater value. Correspondence must not be overlooked, the character of the handwriting, capital letters, underlined words, often throw much light upon an action which appears incomprehensible.

Isolated hallucination, unexpectedly developed, often shows itself with symptoms that enlighten the investigator. There is something wild, unusual, restless, rapid in the eyes, the speech, the gestures, the actions, which indicates that the person is not in his normal state. Nearly always the functions work badly. These facts show that it is not easy to simulate madness and hallucinations.

If doubt exists, isolation must be insisted on, and nearly always, as in the observations of M. Bourne, after a more or less lengthy detention, evident symptoms of insanity dissipate all uncertainty.

Thoughts of ruin, of persecution, &c., have several times been the cause of a class of suicide difficult to prevent. The hallucinated, who are under the impression of these menaces, step by step reduce the quantity of their food. Three of them persisted in doing this for six months. Every patient of this kind, who was not attended to from the first, died in a state of skeleton-like thinness.

A man employed in a tobacco manufactory began by reproaching himself for embezzlement. He struggles against this idea, but it will not quit him: he thinks, then, that he sees at every instant the police around him, who come to hurry him away to the scaffold. Wishing to spare his wife this shame, he remained an entire night while she was asleep with the razor at his throat. Fortunately the thought changed; perhaps he yielded to a gleam of reason, to an instinctive movement of affection; he threw away the deadly instrument. The next day he was brought to the establishment in which I was physician. For two days he had been unceasingly pursued by the same vision. I had just quitted him, when in about a quarter of an hour he was found drowned in a small garden tub, from which he was extricated with difficulty. If this man had cut his wife's throat, and killed himself afterwards, the cause of this fearful tragedy would have been attributed to any but the right motives.

Accusations of theft, of abuse of confidence, of perjury, and voices addressed to the hallucinated have frequently led to avowals from them. "It is true," they admit. Fresh facts I have to add to those we have published to prove that remorse may be a determinating cause of madness and hallucination. A tradesman who until then had deserved the esteem of all who knew him, heard voices reproaching him with a bad action. These voices left him no moment of repose: his family and friends were prodigal of consolation. I was called in, and tried to tranquillize him; everything denoted coming calmness. He went up-stairs to go to bed. A few minutes afterwards he was found hung.

Among the hallucinations which occasion suicide, those must not be forgotten which take a religious shape; such as apparitions of the devil, of the flames of hell, of voices which speak of sins committed, of damnation. We have attended several of these unfortunate persons, among others a Greek lady, who fancied herself condemned to eternal fire; their lamentations, their cries, their howls, will never be effaced from our memory. One fact especially struck us—the horror they felt towards churches; violence would have been necessary to make them enter one. Many of these lunatics would neither fulfil their religious duties nor pray.

If, in the majority of cases, mournful impressions are the cause of suicide, it sometimes happens that the melancholic monomaniacs kill themselves when there is no connexion between cause and effect. Thus many persons, suffering from hallucinations, have thrown themselves out of window, because a voice called them. A lady who fancied she had a serpent in her stomach wished to extract it by means of an incision made with a pair of scissors she begged to have lent to her.

Lunatics are not only dangerous and hurtful to themselves, but equally so to others. Long would be the list of spoliation, ruin, and murder of which they have been the authors. A short time since, in my note, "Sur la perversion des facultés morales et affectives dans la période prodomique de la paralysie génerale,"* I cited the case of a lunatic who had just lost 800,000 francs, ruined his wife, and left five children to the charge of his father-in-law. The action, in connexion with the lunatic who blew out his brains after having torn and thrown into the fire thirty-four notes of a thousand francs each to prevent his wife from inheriting them, is still fresh in the memory. Quite recently I made many useless attempts with MM. Parchappe and Baillarger to

* A. Brierre de Boismont, "Etudes Médico-légales sur la Perversion des Facultés Morales et Affectives dans la Période prodomique de la Paralysie générale." Lues à l'Institut de France, le 24 Décembre, 1860.—*Annales d'Hygiène et de Médecine Légale,* 2e série, tom. xiv., p. 224. 1860.

learn from one of my patients where he had hidden his deeds and his money.

The acts comprehended in the second section are to the number of fifty-two. Of these many consist in menaces or attempts which the originators, according to the legal expression, could not accomplish, owing to circumstances independent of their will, prevented as they were by confinement and surveillance.

In order clearly to understand the slight barrier which separates the ideas of delirium from their execution, we will analyse ten cases, in which are carefully noted the orders given by voices to the hallucinated. They are commanded to do such and such things; to go to the right or to the left; to go out without motive, and while thoroughly appreciating the inutility of these orders are forced to obey. A very pretty, gentle, and well-educated young girl, of religious principles, heard a voice which told her to quit her home. For several days she could not be found. It was vaguely known that she had taken refuge in a forest. These flights having occurred three times, her family, in very natural alarm, brought her to my establishment. Her condition improved. She was able to return home, but another disappearance necessitated fresh restraint; she is now mad. This wandering mania is common enough; it is especially observed among melancholic monomaniacs, who believe themselves pursued by their enemies, think they are being poisoned, &c. One of these frequently changed his lodgings to escape these imaginary attempts, and took his meals every day in the most remote eating-houses—some eat only what they themselves have bought or obtained by stealth.*

Voices compel the hallucinated to keep silence, to repeat their words, to climb trees,† to puff and blow instead of reading; and if observations are addressed to them on this conduct, they answer that they are compelled to act thus.

In place of these orders, futile, ridiculous, and without importance, the voices command reprehensible, dangerous, and criminal actions.

A rich man who lived alone in a house was obliged to sell it; he was completely ruined. Some time afterwards this man, who had long suffered from hallucinations, had a lucid interval, in which he declared he had sold all his property, and thrown the money into the well of his former dwelling, at the instigation of voices which commanded him to do so—(a fact already cited and communicated by Dr. Baron, physician to the Enfants de France).

---

* See chapter on Melancholic Monomania.

† We are citing special facts; it will be obvious that these determinations may vary infinitely.

A clerk, about thirty years of age, was brought eighteen years ago to my establishment in the Rue Neuve St. Géneviève. It was suspected that he was simulating insanity. The house in which he was employed had discovered an embezzlement of about twelve thousand francs, respecting which he could not or would not give any information. Three hours after his arrival he threw into the fire a set of chimney ornaments. I asked him what had induced him to commit such a foolish action; he was some time before replying to me; then he said in a low voice and in a mysterious manner, "He commanded me to do it." From that moment it was impossible to get a word out of him, and he ended by falling into a state of complete insanity.

It is by no means rare for voices to impose upon the insane complete dumbness. There was one in our establishment who was seven years without speaking; and we have another who for two years has declined to say anything to us.

The conviction of the hallucinated as to the reality of these voices will explain these acts. It must be remarked that they yield to a superior strength, as the replies of the majority demonstrate in the most conclusive manner. It is difficult, in fact, to believe that the patient we saw in consultation with MM. Michon and Moreau of Tours, who puffed when we begged him to read, and said he could not help it, would not, under the same impulse, have set fire to his room, or struck down one of us, as he afterwards did.

The acts committed by hallucinated lunatics are often the consequence of an order they receive through the voice of an invisible being.

A cavalry officer, whom we had for twenty years in our establishment, and who was a most gentlemanly man, but full of eccentricity, had killed, under the influence of one of these hallucinations, his colonel at the head of his regiment.

Dr. Botten, formerly physician-in-chief to the Antiquaille Hospital at Lyons, relates, in his essay upon hallucinations, the case of a melancholic monomaniac, several years in that establishment, who had strangled his daughter in obedience to a voice which commanded him to suspend her respiration.

The following fact is in the *Journal d'Hufeland*:—"A Prussian peasant thought he saw and heard an angel which ordered him in the name of God to sacrifice his son upon a pile. He immediately ordered the son to take some wood to a certain spot. The son executed the order; his father laid him upon the pile and killed him. He was the only son!"

A man heard one night an internal voice which said to him:—"You must now kill your child." He rose, resisted the horrible thought, and went to bed again. Scarcely two or three minutes had elapsed when something unknown repeated to him more imperatively than at first, "Instantly kill your child." Arming himself with a hatchet he killed the poor little one.

In an examination that took place some time afterwards, he declared that twice before he had had the horrible idea of killing his son, and that in one of these crises the internal voice said to him, "It is in vain to resist, the boy must die, you must kill him." This thought made him tremble; he prayed, he occupied himself with various labours, and succeeded in discarding the deadly idea which absorbed him. This man declared that for several weeks he had not been intoxicated, and that he was not in that state during the third attack which cost the child its life.*

It is important to remark, indeed, that hallucinations and illusions form one of the most characteristic symptoms of the action of intoxicating drinks. In the numberless cases of suicide and of homicide determined by this cause, that we have related in our *Traité du Suicide et de la Folie Suicide* there were several which had for starting-point the hallucination of hearing.

The lunatic who some years ago killed Dr. Geoffrey, chief physician of the Avignon Asylum, was epileptic and subject to hallucinations; several days before the murder he heard a voice which said to him, "Kill the doctor; if you don't, you'll be unlucky." His conduct established in the clearest manner that he had contrived his plans and acted with judgment, facts of which we have repeated proofs. When the doctor came, he complained of a pain in his foot, begged him to examine it, and while the medical man was stooping, seized him round the body, and plunged into his left side a piece of iron that he had sharpened some days before for this purpose. Although it was certain that he had meditated upon his project, and waited for a favourable moment to put it into execution, his antecedents and the examination left no doubt as to the derangement of his faculties and his continuous state of madness: he was not, therefore, brought to trial. These acts sometimes take place when they are utterly unexpected. A patient who had just been speaking with us, and who appeared very calm, saw his wife enter, whom he believed in connivance with the director of his administration and the employés; he received her with a smiling face, then suddenly raised his arm and struck her twice with the blade of a knife he had hidden: fortunately the whalebone of her stays obviated ill effects.

Some lunatics, tormented by their hallucinations, fall into such a state of despair, that they wish to put an end to their existence; others believe that these machinations are the work of persons

---

* Marc, *De la Folie Judiciaire*, tom. ii., p. 618. Hencke, *Annales*, viii., supplément, p. 186. Caspar, *Traité Pratique de Médecine Légale*, 1er vol.

near, conceive a hatred towards them and seek revenge. One of our patients rushed upon her husband, seized him by the throat, and it was with great difficulty she was prevented from strangling him. An old lady, persuaded that her servants wished to steal her ear-rings, armed herself with scissors to resist their attacks. A lunatic who thought he was being poisoned and had seen the poison put into his food, declared he would kill some one, and finished by naming a real person.

Hallucinations are not only the cause of dangerous actions, suicide, and murder, but also of theft, incendiarism, &c. These determinations are specially due to voices which command the strangest things.

One of our lunatics seized upon anything which fell into his way. He was well bred and of a good fortune. He committed his larcenies with marvellous dexterity, and was accordingly constantly watched. Every instant it was necessary to search him, and articles were found hidden in various parts of his clothes. When reproached for this incredible mania, he replied: "I am told to take all these things because they belong to me." This disease was developed to such an extent in the case of a lunatic we saw some time ago at the Sainte Gemme asylum, near Angers, when we went there to observe some cases of pellagra, that it was necessary to put him in a strait waistcoat. Despite this precaution, he tried to steal something from us.

A person suffering from hallucinations confided to our care heard a voice, which told him to set fire to his chamber, and he acted as commanded.

Jonathan Martin, the new Erostratus, who set fire to York Cathedral, said to the judge when questioned: "Your charge of theft is ridiculous, and you do well to give it up; I never intended to abstract anything; but *an angel having ordered me* by the will of God to set fire to the church, I was obliged to furnish proofs that I alone had done this deed, so that no one else should reap the honour, or, if you like it better, bear the punishment." Jonathan Martin, declared a lunatic, was confined in Bethlehem.

A young girl of less than fifteen, named Grabowska, afflicted with nostolgia, twice set fire to the house, in order to quit her masters. She declared that from the moment she entered their service she was unceasingly possessed with the desire to burn the place. It seemed to her that a spectre continually before her impelled her to this act. It was noticed that this girl for a long time suffered from violent headache, and that menstruation was behindhand.*

The illusions of sight bearing upon the changes of persons and things are so important, that although we have several times

* Marc, *Mémoire sur la Pyromanie,* tome ii., p. 356. Klein, vol. ix., *Annales Judiciaires.*

insisted upon this symptom, all our efforts ought to be employed in demonstrating their frequency and their results. It is no question here of reasoning, more or less erroneous, that is declared to be easily appreciable by every man of judgment, but of a sensorial disorder well known to specialistic doctors, which seizes half, sometimes, perhaps, three-fourths, of the insane, and the evidence of which in their eyes is such that even the voice of kindred is without strength to triumph over this illusion. Among other examples we have under our eyes, the following has left an ineffaceable impression:—A lady afflicted with melancholy, whose case we have elsewhere cited, asked every day in the most pathetic tone and the most heart-rending accents, to see her husband and her son. She would not take any food, and had to be fed with the œsophagus tube. I learnt that the same complaints had been uttered in another establishment, and that the meeting so ardently desired produced no effect. Nevertheless, touched like others in the house by a grief which appeared so truthful I sent for the husband and son: despite my experience I still hoped. After having seen them the poor lady heaved a profound sigh, and cried, "It is not those!" The trial was made a second time without more success; it was not repeated, for it might have led to sad consequences for the child. Five years after these two attempts the patient unceasingly repeated—"I implore you not to separate an unhappy woman from her child and her husband!"

(*To be continued.*)

---

